# The Utility of Blood and Bone Marrow Films and Trephine Biopsy Sections in the Diagnosis of Parasitic Infections

**DOI:** 10.4084/MJHID.2015.039

**Published:** 2015-06-01

**Authors:** Clare E. Miller, Barbara J. Bain

**Affiliations:** 1 Honorary Clinical Research Fellow, Centre for Haematology, 5^th^ Floor, Commonwealth Building, Hammersmith Hospital campus of Imperial College London, Hammersmith Hospital, 150 Du Cane road, London W12 0HS, UK.; 2 St Mary’s Hospital campus of Imperial College London, St Mary’s Hospital, Praed Street, London W2 1NY, UK.

## Abstract

The laboratory haematologist has a role in the diagnosis of parasitic infections. Peripheral blood examination is critical in the diagnosis of malaria, babesiosis, filariasis and trypanosomiasis. Bone marrow examination is important in the diagnosis of leishmaniasis and occasionally leads to the diagnosis of other parasitic infections. The detection of eosinophilia or iron deficiency anaemia can alert the laboratory haematologist or physician to the possibility of parasitic infection. In addition to morphological skills, an adequate clinical history is important for speedy and accurate diagnosis, particularly in non-endemic areas.

## Introduction

Microscopic assessment of blood films and bone marrow samples plays a key role in the diagnosis of several parasitic infections. Some organisms e.g. malaria parasites, babesiae, trypanosomes*,* leishmaniae and microfilariae, may be directly visualised in the blood film or marrow, or associated abnormalities such as thrombocytopenia, eosinophilia or compensatory bone marrow changes may provide diagnostic clues. Iron deficiency anaemia can be seen as a result of blood loss from the gastrointestinal tract with chronic parasitic infections of the bowel, or from the urinary bladder with chronic schistosomiasis. Skill is required to detect and accurately differentiate organisms, particularly when they are scanty. Concentration techniques such as buffy coat preparation, centrifugation and filtration can be used to enhance sensitivity. Serological assays are available for a number of infections, but these should be used as an adjunct to microscopy, as none is sensitive or specific enough to be used on its own to establish a diagnosis.^1^ It is important that the laboratory is informed if there is clinical suspicion of a parasitic infection, including details of any relevant travel history, in order to ensure optimal slide preparation and a high index of suspicion on examining the slides.

## Peripheral Blood Films

### Malaria

Examination of thick and thin blood films remains the primary method of diagnosis of malaria in most clinical laboratories. It is recommended as the diagnostic method of choice where the facilities and expertise are available, particularly in cases of severe malaria.^2^ Compared with the rapid diagnostic tests (RDTs) which detect parasite-specific antigens or enzymes, it has the advantage of allowing species to be determined and parasites to be quantified and may help identify other causes of fever. Delays in the diagnosis of malaria often occur due to the diagnosis not being considered promptly; non-specific laboratory clues include elevated lactate dehydrogenase, presence of atypical lymphocytes, elevated aspartate transaminase and thrombocytopenia.^3^

#### Film preparation

A thick film is preferable for detection of parasites and a thin film for species identification. Although malarial parasites may be detected in May–Grünwald–Giemsa-stained blood films, the specific parasite and erythrocyte features are more distinguishable at higher pH with Leishman or Giemsa staining; a rapid Field stain may also be used. Blood films should be prepared no longer than three to four hours after blood collection to minimise the risk of distorted morphology and the potential appearance of parasite stages not normally occurring in the blood.^4^

#### Parasite and Erythrocyte Morphology

**Figure 1:**
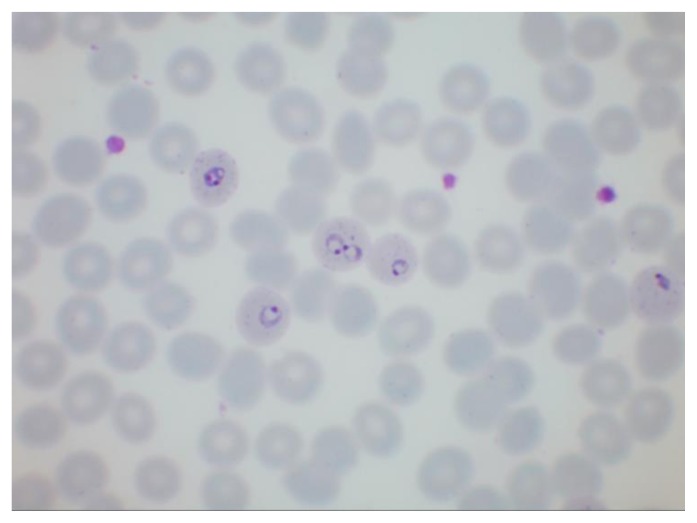
Thin film showing five non-enlarged erythrocytes parasitised by ring forms of *Plasmodium falciparum*. Note the presence of Maurer’s clefts and a cell containing two parasites

**Figure 2:**
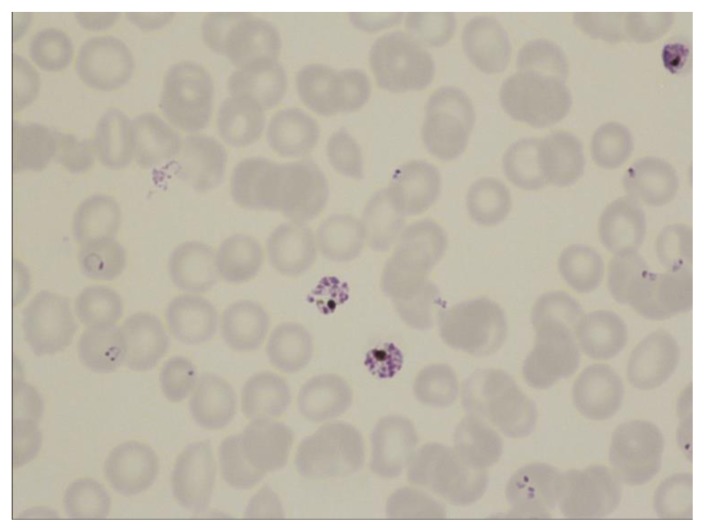
Thin film in *P. falciparum* infection showing numerous ring trophozoites and two schizonts. Note one ring form with a double chromatin dot and one accolé (shoulder) form. The observation of schizonts in the blood is uncommon but they are sometimes seen in heavy infections.

**Figure 3:**
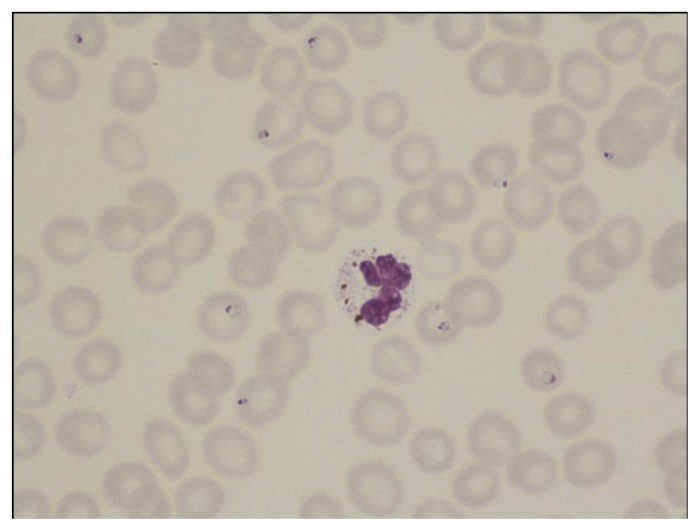
Thin film in *P. falciparum* infection showing ring forms and a neutrophil containing malaria pigment.

**Figure 4:**
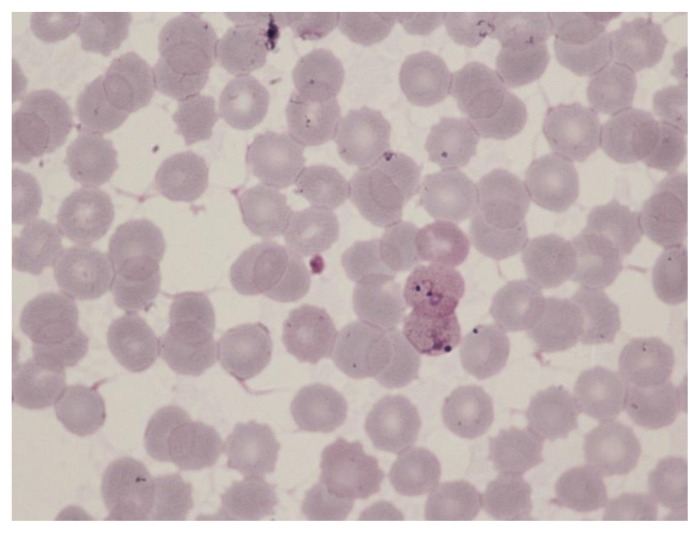
Thin film in *P. vivax* infection showing two amoeboid trophozoites in enlarged erythrocytes. Schüffner’s dots are apparent.

**Figure 5:**
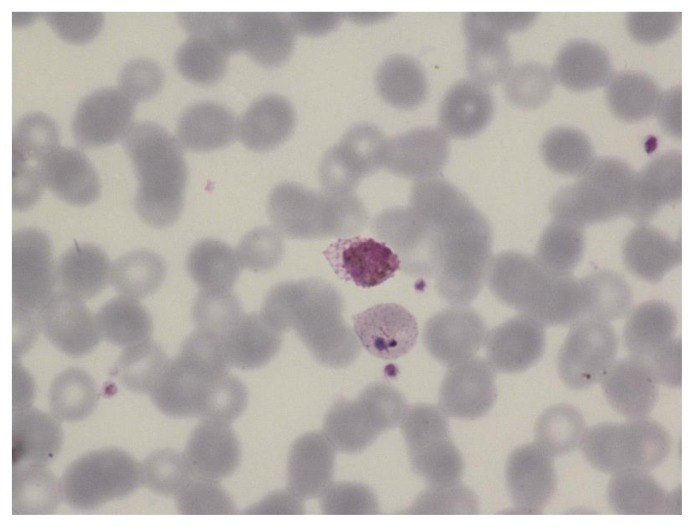
Thin film in *P. vivax* infection showing a trophozoite and a gametocyte. The erythrocytes are enlarged and Schüffner’s dots are apparent.

**Figure 6:**
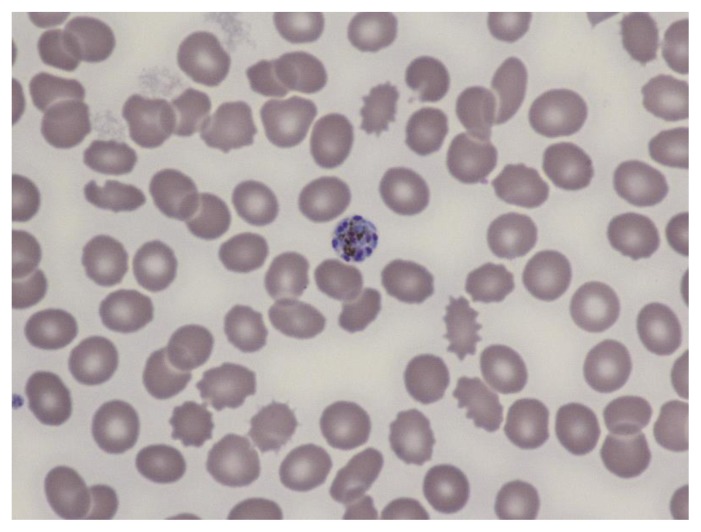
Thin film in *P. ovale* infection showing a large trophozoite in an enlarged erythrocyte with prominent Schüffner’s dots.

**Figure 7:**
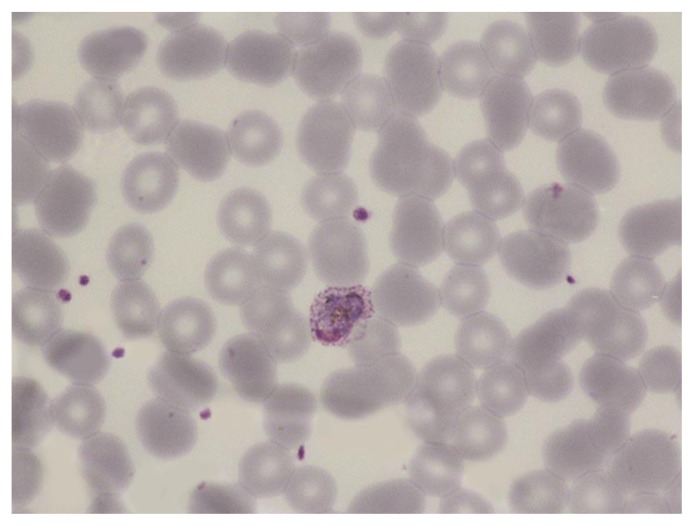
Thin film in *P. malariae* infection showing a schizonts within a non-enlarged erythrocyte.

The distinguishing parasite and erythrocyte features permitting identification of the different plasmodium species are well established (Figures 1–7, for morphology of *Plasmodium knowlesi* and for comprehensive images of other species see references 4 and 5). It should be noted that *Plasmodium knowlesi*, a parasite only occasionally introduced into Europe, can have some parasites that resemble *P. falciparum* and others that resemble *P. malariae*.

Accurate laboratory diagnosis of malaria is essential, particularly to recognise potentially fatal *P. falciparum* infection*.* Malarial parasites typically appear as cytoplasmic inclusions within erythrocytes; phagocytosed merozoites and sometimes schizonts within neutrophils may be seen in *P. falciparum* with a high parasitaemia.^6^,^7^ Parasitized red cells have an altered appearance, the nature of which varies according to the implicated species; cells are typically enlarged in *P. vivax* and *P. ovale* infections (Figures 5, 6 and 7). The malarial pigment, haemozoin, is a degradation product of haemoglobin and may be seen in monocytes and occasionally neutrophils (Figure 3). It can be visualised readily in stained or unstained films and is birefringent when polarised light is used.^8^ Monocytes containing malarial pigment can often be found in the blood for many days after parasitized red cells have disappeared; this can be useful in making a retrospective diagnosis of malaria.^9^ In the case of *P. falciparum* or *P. knowlesi* infection the degree of parasitaemia should be reported to help assess disease severity and monitor treatment response. A count of the proportion of cells that are parasitized can be made, facilitated by a Miller graticule, or the number of parasites per ml can be calculated in relation to the number of white cells. Paradoxically, patients with few or no parasites detectable on initial blood examination may in fact be seriously ill due to parasitized red cells being sequestered in tissues. Parasitaemia is frequently over- or under-estimated and participation in quality assessment schemes and appropriate referrals to reference laboratories are important measures to improve practice.^10^,^11^ All films should be examined by two people, at least one of whom should have considerable experience in the field. For laboratories that do not often see cases of malaria, examination of films can usefully be supplemented by RDTs.

#### Associated abnormalities

The differential blood count varies considerably between individuals with malaria. Thrombocytopenia is seen in approximately 60–80% of people, most commonly but not only in those with *P. falciparum* or *P. knowlesi* infections.^12^–^14^ Possible causes include reduced platelet survival from peripheral destruction, enhanced splenic uptake or sequestration, and decreased platelet production.^15^ Complicating disseminated intravascular coagulation can occur in falciparum malaria and, rarely, in vivax malaria.^16^ Other potential findings include a haemolytic anaemia, leucocytosis or leucopenia, early neutrophilia (with *P. falciparum*) or neutropenia, lymphocytosis or lymphopenia (more commonly lymphopenia) and monocytosis or monocytopenia. Worse prognosis has been associated with both lymphopenia and lymphocytosis in different studies. In one study in children a high lymphocyte count and a low monocyte count were found to correlate with mortality but thrombocytopenia did not.^7^ In a second study, thrombocytopenia, leucocytosis and neutrophilia were significantly associated with severe falciparum malaria in comparison with non-severe and non-falciparum malaria but the lymphocyte count and the neutrophil:lymphocyte ratio did not differ between groups; the neutrophil: lymphocyte ratio did, however, correlate with the degree of parasitaemia.^17^ In a third study severe malaria was associated with a higher neutrophil:lymphocyte ratio, a lower lymphocyte count and a lower monocyte count than non-severe malaria.^18^ In view of the conflicting results in these and other studies of leucocyte counts, such changes cannot be regarded as reliable indicators of disease severity. The reticulocyte count may be inappropriately low as result of bone marrow suppression; pancytopenia has also been reported.^19^ Clinical and laboratory staff should also be alert to the possibility of a severe delayed haemolytic anaemia in patients who are treated with artemisinin.^20^ Atypical lymphocytes are present in malaria and in some patients with hyper-reactive malarial splenomegaly.

### Babesiosis

Babesiosis is an uncommon tick-borne parasitic disease caused by a haematoprotozoan of the genus Babesia. *Babesia microti* is the commonest causative organism and is endemic in southern New England, southern New York state, Wisconsin and Minnesota, primarily occurring between May and October. It is more often detected in hyposplenic and immunosuppressed patients and the parasitaemia levels are also usually higher in these patient groups. It is an emerging threat in transfusion medicine in the United States, with 162 reported transfusion-associated infections between 1982 and 2013 and 12 associated fatalities in the period 2005–2008.^21^ *B. duncani* has also been transmitted by transfusion.^22^ Transfusion-transmitted infection, like naturally occurring tick-transmitted infection, is more often recognised in hyposplenic patients including patients with sickle cell disease.^23^ *B. divergens*, a parasite of cattle, causes sporadic cases of babesiosis in the USA, Europe and Asia, most often in hyposplenic patients. *B. bovis* infection also occurs occasionally in Europe.^24^ *B. venatorum*, a parasite of roe deer, causes occasional cases in Europe.

#### Film preparation

Thick and thin films should be examined as for malaria.

#### Parasite Morphology

The trophozoites of Babesia species are small rings, easily confused with those of *P. falciparum*. They are 1–5 μm in diameter with one, two or three chromatin dots and scanty cytoplasm. Sometimes they are pyriform (pear-shaped) and either paired or have the pointed ends of four parasites in contact to give a characteristic Maltese cross formation (see reference 5). Extracellular parasites may be seen and can form clusters.^24^,^25^ *B. microti* and *B. duncani* trophozoites are indistinguishable morphologically; both are associated with Maltese cross and ring forms, the latter with small to large cytoplasmic vacuoles. Their smaller size, vacuolation, polymorphism of the ring forms, the presence of trophozoites and absence of haemozoin all help distinguish them from *P. falciparum.* Malaria RDTs are negative in babesiosis. *B. divergens* and *B. venatorum* typically appear as pyriform pairs of parasites at the periphery of the erythrocyte but also appear, rarely, as tetrads.^22^,^26^

#### Associated abnormalities

Babesiosis is often associated with lymphopenia and thrombocytopenia. Haemolysis is usually mild. There may be atypical lymphocytes.

### Trypanosomiasis

African trypanosomiasis (sleeping sickness) is caused by *Trypanosoma brucei gambiense* (West Africa and western Central Africa) and *T. brucei rhodesiense* (East, Central and Southern Africa). It is transmitted by the tsetse fly. American trypanosomiasis (Chagas’ disease) is caused by *T. cruzi*. Trypanosomes may be detected in the peripheral blood as extracellular parasites (trypomastigotes). As with malaria, the quality of blood film microscopy is improved by participation in external quality assessments.^27^

#### Film preparation and staining

Trypanasomes may be seen moving in a wet preparation when a drop of anticoagulated blood is placed on a slide, beneath a coverslip, for microscopic examination. They can also be detected in fixed preparations such as thick or thin films or buffy coat films. Scanty parasites are more readily detected by examining the sediment of 10–20 ml of haemolysed blood. Repeated examinations and concentration techniques may be needed, particularly for *T. brucei gambiense* and *T. cruzi.* Preparations should be examined within four hours of sampling. Live trypanosomes are highly infectious and appropriate laboratory standard precautions must be adhered to when handling specimens.

#### Parasite morphology

**Figure 8:**
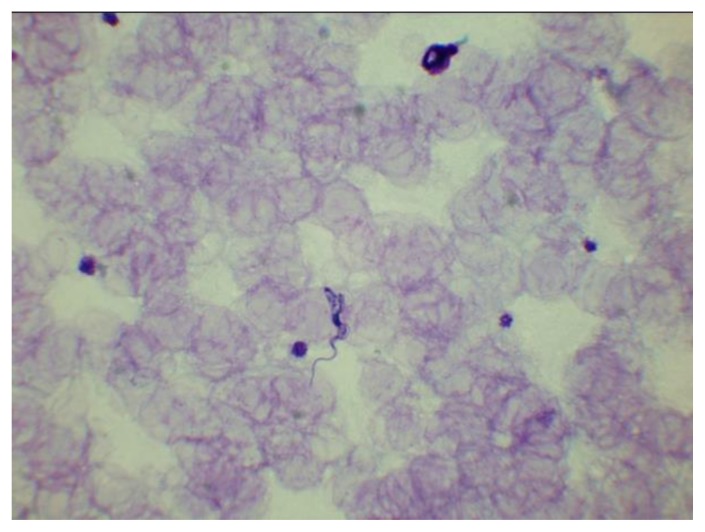
A thick film showing a trypomastigote of *T. brucei rhodesiense*. *T. brucei gambiense* is morphologically identical.

*T. brucei gambiense* and *T. brucei rhodesiense* are morphologically indistinguishable, though the latter are more readily detectable in blood films. They are 13–42 μm long with a slender body, a centrally placed nucleus, a dot-like kinetoplast and a single flagellum (Figure 8). The flagellum is joined to the body by an undulating membrane and is crucial for parasite motility, transmission and pathogenesis.^28^ *T. cruzi* parasites measure 12–30 μm and have a larger kinetoplast than the African trypanosomes. They can be distinguished morphologically from *T. rangeli* which has a similar geographical distribution.

#### Associated features

Normocytic normochromic anaemia and thrombocytopenia are often seen with African trypanosomiasis.^29^ Lymphocytosis and mild anaemia may be observed in the acute phase of Chagas’ disease.

### Filariasis

Filariasis affects over 120 million people worldwide and is endemic in 80 countries.^30^ Lymphatic filariasis is caused by one of three nematodes (*Wuchereria bancrofti, Brugia malayi* and *Brugia timori*, the latter confined to part of Indonesia); filarial infection of the subcutaneous tissues is caused by *Loa Loa*. The larvae of these worms, the microfilariae, are transmitted by mosquitoes to humans, where they can be found in the blood and show periodicity. *W. bancrofti* and *B. malayi* typically release their microfilariae at night, whereas those of *Loa loa* are released during the day.

#### Film preparation

Wet preparations of blood or buffy coat samples may be used for detection of parasites; examination of a stained film (Giemsa or another appropriate stain) is needed for determining species. Concentration methods using centrifugation or stained polycarbonate filters may enhance detection.^1^

#### Parasite morphology

**Figure 9:**
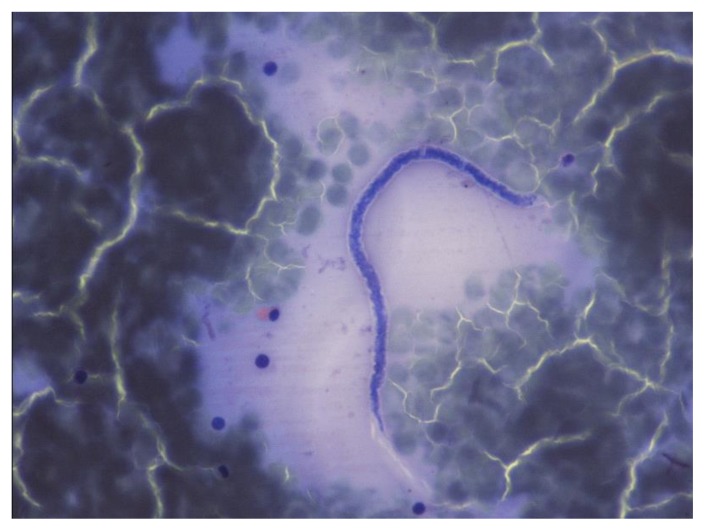
Microfilaria of *Wuchereria bancrofti* in a thick film.

**Figure 10:**
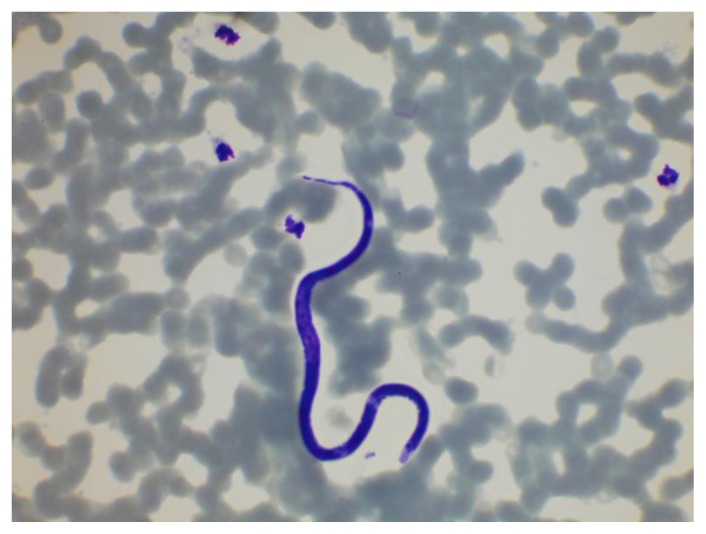
Microfilariae of *Loa loa* in a thin film stained with a May–Grünwald–Giemsa stain. Note that the nuclei extend into the tail.

**Figure 11:**
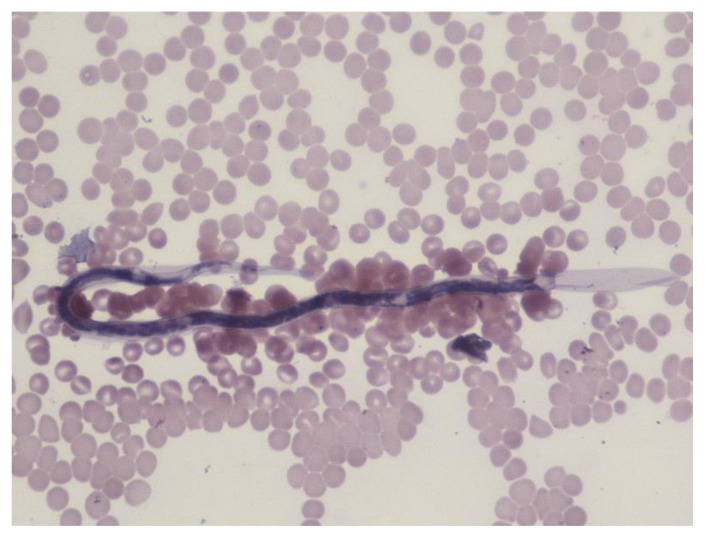
Microfilaria of *Loa loa* in a thin film stained with Giemsa and Dellafield stain, which shows the sheath well.

Microfilariae are classified on the basis of body length and width, the presence or absence of a sheath, derived from remnants of the egg membrane, the number of nuclei in the body and the appearance of the tail including the presence or absence of nuclei in the tail tip (Figures 9–11). In general, pathogenic filariae are sheathed and non-pathogenic are non-sheathed. However, *B. malayi* is sometimes seen unsheathed.^31^ *Onchocerca volvulus*, which infects subcutaneous tissues (adult forms) and the eyes (microfilariae), is occasionally seen in the blood, especially in heavy infections and after therapy; it is unsheathed with a pointed tail that lacks nuclei.^31^

#### Associated features

Lymphatic filariasis is typically associated with an eosinophilia; blood eosinophil count may be used as a nonspecific screening tool in endemic areas.^32^

### Others

Rarely, *Toxoplasma gondii* has been identified in the peripheral blood, either extracellularly or within neutrophils, in patients with toxoplasmosis and underlying immune deficiency.^33^,^34^ Phagocytosed leishmaniae (amastigotes) are occasionally detectable within peripheral blood monocytes or neutrophils, particularly in immunosuppressed subjects. There may be an associated pancytopenia, anaemia, leucopenia or thrombocytopenia; red cell agglutination, fragmentation and rouleaux are also seen.^35^

## Bone Marrow Cytology

### Malaria

Malarial parasites may be visualised in red cells or neutrophils or apparently free in a bone marrow aspirate (see reference 36), although bone marrow aspiration is not a recommended diagnostic method for suspected malaria. In acute falciparum malaria the bone marrow may be hypocellular, normocellular or mildly hypercellular. Immature gametocytes, which are not usually seen in the peripheral blood, may be detected in the bone marrow.^37^,^38^ In chronic falciparum malaria there is hypercellularity with erythroid hyperplasia. Other features include dyserythropoiesis, giant metamyelocytes and increased eosinophils, lymphocytes, plasma cells and macrophages, sometimes with haemophagocytosis.^6^,^39^,^40^ The bone marrow in *P. vivax* malaria is also characterized by dyserythropoiesis, increased macrophages (some showing haemophagocytosis), increased plasma cells and sometimes increased eosinophils.^6^ In hyper-reactive malarial splenomegaly there may be a marked increase in bone marrow lymphocytes.^41^

### Babesiosis

Haemophagocytosis has been observed in the bone marrow in babesiosis.^42^,^43^

### Leishmaniasis

Visceral leishmaniasis is a vector-borne protozoan disease associated with replication of parasites in macrophages; it is transmitted by female sandflies. Bone marrow aspiration is very useful in the diagnosis of visceral leishmaniasis and is a recommended diagnostic method when this is suspected. Leishmaniasis usually results from *Leishmania donovani* in the Indian subcontinent, Asia and Africa (in adults and children) or from *L. infantum* in the Mediterranean region and southwest and central Asia; in South America this same species is known as *L. chagasi*, infection being seen primarily in young children and immunosuppressed individuals. Other species e.g. *L. tropica* in the middle east and *L. amazonensis* in South America are occasionally viscerotropic; all may be detected by bone marrow examination.^44^,^45^ Leishmaniasis is increasingly been seen in the context of HIV co-infection and generally represents reactivation of previously subclinical infection.^45^ It is occasionally seen as a cause of pancytopenia even in patients living outside areas of endemicity and without a specific travel history.^46^,^47^

**Figure 12:**
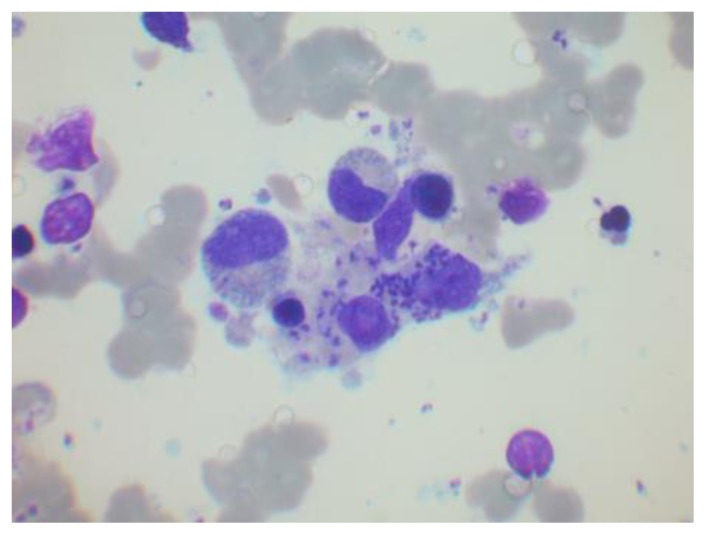
Bone marrow aspirate film showing a macrophage containing Leishman–Donovan bodies. There are also some apparently extracellular organism.

Diagnostic sensitivity for splenic, bone marrow and lymph node aspirate smears is >95%, 55–97% and 60% respectively.^45^,^48^ Aspirate films can be stained with a Giemsa, May-Grünwald-Giemsa or Leishman stain. Amastigote forms, called LD bodies, may be visualised; they are characterised by a small paranuclear rod-like body known as the kinetoplast, giving the organism a characteristic ‘double-dot’ appearance (Figure 12). Leishmaniae are obligatory intracellular parasites of mononuclear phagocytes, but they may appear more abundant extracellularly due to disruption of macrophages during spreading of aspirate films.^49^ Increased macrophages, plasmacytosis and erythroid hyperplasia are seen in the majority of cases.^35^ Dyserythropoiesis can be striking, to the extent that misdiagnosis as myelodysplastic syndrome has occurred when the parasites have been overlooked.^50^,^51^ Plasma cells (including Mott cells and cells containing crystals or Russell bodies), dysmyelopoiesis, free floating cytoplasm and intracellular LD bodies in cells other than histiocytes (polymorphs, metamyelocytes) are uncommon features.^35^,^52^ Increased eosinophils and eosinophilic precursors are seen in 15–27% of cases.^35^,^53^ There is associated haemophagocytosis in up to 75% of individuals; diagnosis can be challenging due to overlapping clinical features. Aspirates are often reported as negative for LD bodies at disease onset, but in our experience LD bodies may be present but missed because they are infrequent or there was not a high index of suspicion.^54^

### Others

Trypanosomes are sometimes detected in the bone marrow, but less often than leishmaniae. Detection is more common in immunosuppressed patients.^55^,^56^ Microfilaria are occasionally observed (see ref 36), also more commonly in the immunocompromised host; there may be associated marrow hypoplasia.^57^,^58^ Toxoplasma have also sometimes been found in immunodeficient patients, either as free organisms (see ref 36) or within cysts.^59^ An increased number of bone marrow eosinophils and their precursors are often seen with helminth infections.

## Bone Marrow Histology

A bone marrow trephine biopsy is not a recommended method for the diagnosis of parasitic infections but it is necessary to recognise the histological features since this is occasionally an unexpected diagnosis, for example when a biopsy is done to investigate fever, pancytopenia or hepatosplenomegaly.

### Malaria

Bone marrow histology in malaria typically reveals hypercellularity with increased macrophage activity, often with haemophagocytosis. The unstained bone marrow films of patients who have had repeated bouts of malaria may appear slate grey or black because of the accumulation of haemozoin.^6^ It is important to distinguish haemozoin from formalin pigment. Haemozoin may be seen not only in macrophages but also within erythroid and granulocytic precursors, possibly contributing to dyserythropoiesis and erythroid suppression.^60^ There is correlation between the amount of haemozoin deposition and the severity of anaemia in children with *P. falciparum* infection.^61^ During attacks of acute malaria, sinusoids may be packed with parasitized red cells.^39^ Gametocytes at different maturation stages can be identified in haematoxylin and eosin (H&E)-stained sections, progressing from immature leaf-shaped forms to mature forms with a more crescentic shape.^62^ The majority of immature gametocytes may be observed in extravascular spaces, whilst most mature gametocytes are typically seen in intravascular spaces.^62^,^63^

### Leishmaniasis

The bone marrow is hypercellular in the majority of cases of visceral leishmaniasis; numerous LD bodies are typically present and allow the distinction from haematological malignancies which can present with a similar clinical picture. LD bodies appear as 1–3 μm round bodies inside macrophages; their morphological features are often less apparent than when the parasites are visualised in an aspirate. They are sometimes confused with the fungus *Histoplasma capsulatum* in view of their small size. However, leishmaniae fail to stain with periodic acid-Schiff (PAS) or silver stains and an H&E or Giemsa stain will demonstrate the ‘double-dot’ of the nucleus and kinetoplast. Additional findings include necrosis, noncaseating granulomas, increased fibrotic foci and increased vascularity. Estimations of the frequency of these findings have varied considerably between studies, perhaps reflecting differences in average parasite densities between regions.^35^,^52^,^53^,^64^

### Others

Toxoplasma infections may be detected in bone marrow trephine specimens. In immunocompetent individuals the only finding may be of granuloma formation. In immunodeficient patients *T. gondii* organisms are occasionally seen. They usually take the form of tachyzoites, which are 3–6 μm in diameter and have a tiny single nucleus. Occasionally, cysts containing numerous bradyzoites are present. Tachyzoites are negative with a PAS reaction, whereas cysts or bradyzoites are generally well recognised by this staining.^65^ Immunohistochemistry is useful to confirm *T. gondii* infection and to discriminate the parasite from cellular debris.^65^

*T. cruzi* may be detected in trephine biopsy sections from immunosuppressed patients with acute Chagas disease.^56^ Very rarely schistosomal eggs have been observed in a trephine biopsy section.^66^ *Pneumocystis jirovecii* can involve the bone marrow, particularly in immunocompromised hosts, but this organism has now been recognised as a fungus rather than a protozoan.^67^,^68^

## Conclusions

Peripheral blood examination is critical in the diagnosis of malaria, babesiosis, filariasis and trypanosomiasis but it is also important to be aware of the possibility of diagnosis of these infections from bone marrow aspirates or trephine biopsy sections. In the case of leishmaniasis, it is bone marrow examination that is of major diagnostic importance, while organisms are only rarely detected in the peripheral blood.
